# Stage-specific control of stem cell niche architecture in the *Drosophila* testis by the posterior *Hox* gene *Abd-B*

**DOI:** 10.1016/j.csbj.2015.01.001

**Published:** 2015-01-21

**Authors:** Fani Papagiannouli, Ingrid Lohmann

**Affiliations:** Centre for Organismal Studies (COS) Heidelberg, Cell Networks – Cluster of Excellence, University of Heidelberg, D-69120, Germany

**Keywords:** Abd-B, abdominal-B, CySCs, somatic cyst stem cells, ECM, extracellular matrix, GSCs, germline stem cells, SCCs, somatic cyst cells, SGPs, somatic gonadal precursors, wt, wild type, L3, 3rd instar *Drosophila* larvae, Abd-B, *Drosophila* testis, Integrin, Talin, Niche positioning

## Abstract

A fundamental question in biology is how complex structures are maintained after their initial specification. We address this question by reviewing the role of the *Hox* gene Abd-B in *Drosophila* testis organogenesis, which proceeds through embryonic, larval and pupal stages to reach maturation in adult stages. The data presented in this review highlight a cell- and stage-specific function of Abd-B, since the mechanisms regulating stem cell niche positioning and architecture at different stages seem to be different despite the employment of similar factors. In addition to its described role in the male embryonic gonads, sustained activity of Abd-B in the pre-meiotic germline spermatocytes during larval stages is required to maintain the architecture of the stem cell niche by regulating βPS-integrin localization in the neighboring somatic cyst cells. Loss of Abd-B is associated with cell non-autonomous effects within the niche, leading to a dramatic reduction of pre-meiotic cell populations in adult testes. Identification of Abd-B target genes revealed that Abd-B mediates its effects by controlling the activity of the sevenless ligand Boss via its direct targets *Src42A* and *Sec63*. During adult stages, when testis morphogenesis is completed with the addition of the acto-myosin sheath originating from the genital disc, stem cell niche positioning and integrity are regulated by Abd-B activity in the acto-myosin sheath whereas integrin acts in an Abd-B independent way. It seems that the occurrence of new cell types and cell interactions in the course of testis organogenesis made it necessary to adapt the system to the new cellular conditions by reusing the same players for testis stem cell niche positioning in an alternative manner.

## 1 Introduction

*Hox* genes are master regulators of morphogenesis that code for homeodomain-containing transcription factors with a high conservation in different metazoans. Studying their function during embryogenesis in animals as diverse as insects and vertebrates revealed their critical role in establishing the identity of segmental structures along the anterior-posterior (A/P) body axis of these organisms [66]. More recent research emphasizes the role of *Hox* genes as cell-type switches [8,55,79] that control local cell behaviors resulting in the development of segment-specific structures and organs [3,43,66]. *Hox* genes are expressed throughout an animal's life [66], suggesting that they control different aspects of morphogenesis in a stage-dependent manner. However, due to the deleterious effects of *Hox* gene mutations, which normally result in the death of the organism at the end of embryogenesis, later Hox functions have rarely been studied [2,61,62,74]. Even more important, it has not been successfully addressed if and how *Hox* genes control the development and maintenance of structures and organs throughout the life of an organism, from embryogenesis to adulthood when new cell types and interactions emerge in the various stages. To answer this question, we use the fruitfly *Drosophila melanogaster*, a well-established model system with a wealth of available genetic tools for conditional, cell-type and stage-specific knockdown of genes to investigate stem cell function in a highly precise way.

Adult stem cells are the lifetime source of many differentiated cell types that maintain homeostasis of a tissue and respond to injury when challenged. They reside in microenvironments, the stem cell niches, that have an important role in stem cell behavior [85]. The stem cell niche (thereafter referred to simply as the “niche”) relies on a subset of differentiated cells or extracellular substrates that recruits the stem cells and promotes stem cell maintenance in vivo through physical contacts and diffusible signals [107]. In this review we discuss how the *Drosophila* male stem cell niche is maintained after its initial specification, we review the current state of the art on stage-specific niche architecture and function, and explain how the posterior Hox gene *Abd-B* controls, as an upstream regulator, niche positioning and integrity in a cell-type and stage specific way.

## 2 *Drosophila* testis and the male stem cell niche

In all adult tissues harboring stem cells, the stem cell niche has a critical function as an organizer, which recruits the stem cells and provides the microenvironment required for stem cell maintenance. Much of the knowledge we have on testis stem cells and their niche comes from studies in *Drosophila*, a well-characterized system to study the biology of the stem cell niche, the germline stem cells and spermatogenesis [69]. Organogenesis of the *Drosophila* testis, a structure first made by the coalesce of germ cells and somatic gonadal cells at stage 14 of embryogenesis, continues throughout embryonic and larval stages, and goes through a second wave of organ shaping in the pupae, to reach maturation in adult stages. The *Drosophila* male stem cell niche, called the hub, is a cluster of non-dividing cells specified in the anterior most somatic gonadal cells already before gonad coalesce [4,20,21,25,40,53].

**Fig. 1:**
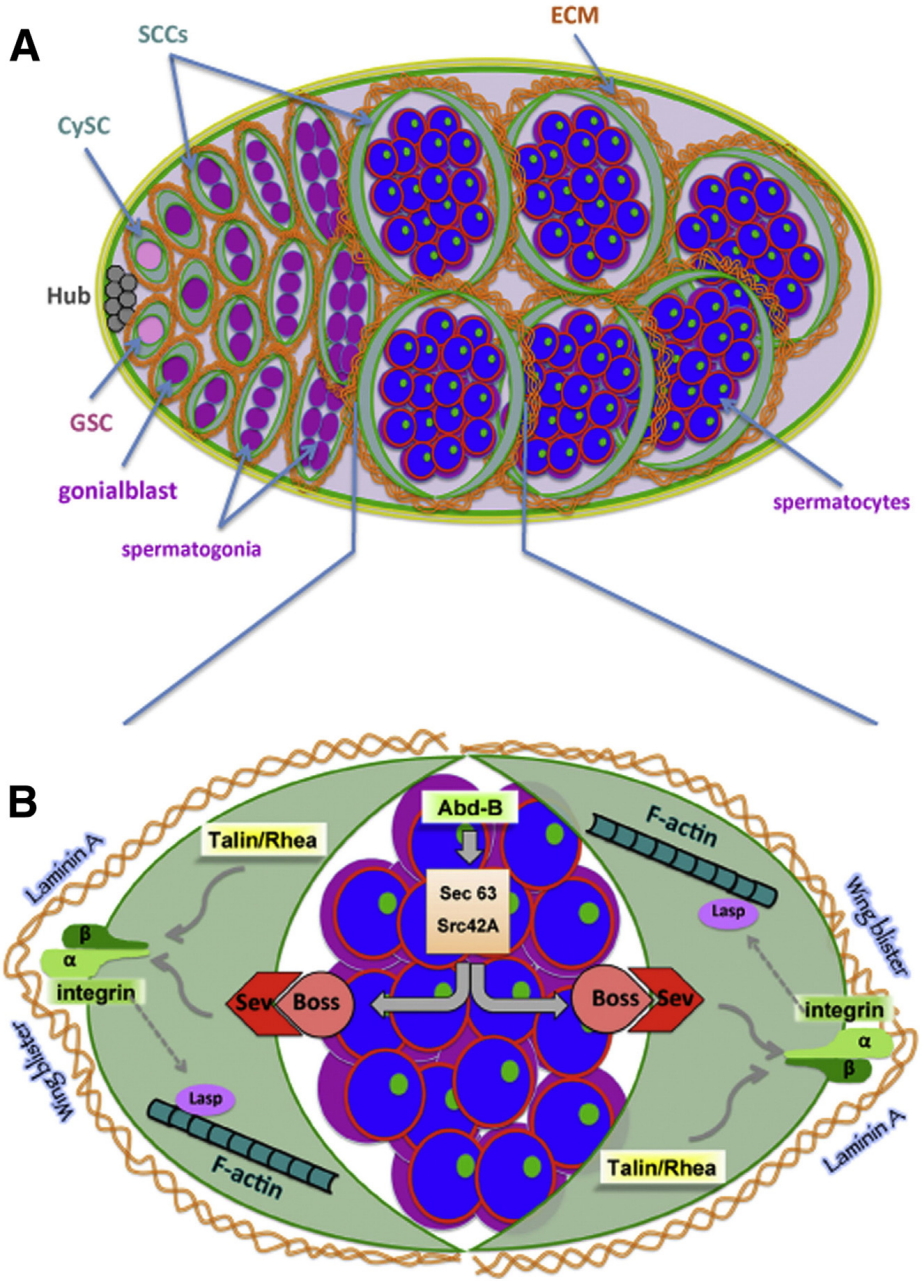
(A) Diagram showing the stem cell niche and early stages of *Drosophila* spermatogenesis. GSC: germline stem cell, CySC: somatic cyst stem cell, SCC: somatic cyst cell, ECM: extra-cellular matrix. Testicular cysts comprise of a pair of cyst cells flanking the germline (GSCs, spermatogonia and spermatocytes). Integrin localization is indicated in green. ECM molecules (orange) surround the cyst cells and testicular cysts. Within the spermatocytes, the red line indicates the nuclear membrane, the green dots resemble Abd-B distribution in the nucleolus and blue represents the nucleus. In our model, stem cell niche positioning and integrity are regulated by several factors like integrins, ECM components and actin filaments that build together a dynamic scaffolding network. Integrins generate tensional forces by interacting with the actin cytoskeleton and ECM to maintain the rigidity of the testicular cysts and keep the testis stem cell niche at the anterior part of the testis by excluding it from the rest of the testis. (B) Diagram showing key players involved in larval stem cell niche positioning. Schematic diagram of a testicular cyst depicting local germline-soma signaling and key players involved in niche positioning.

The first signs of testis organogenesis are already detected in late embryogenesis (stages 14-17), once the specified hub cells recruit the anterior-most germ cells to become the germline stem cells (GSCs) [88]. A testis with a mature stem cell niche and all pre-meiotic stages is detected at 3rd instar larvae (L3) (Fig. 1A). The *Drosophila* testis contains two types of stem cells: the germline stem cells (GSCs) and the somatic cyst stem cells (CySCs). Each GSC is flanked by two somatic cyst stem cells (CySCs) and both types of stem cells are maintained through their association to the hub cells, a cluster of non-dividing cells forming the niche organizer. Upon asymmetric cell division, each GSC produces a new GSC attached to the hub and a distally located gonialblast. The CySCs also divide asymmetrically to generate a CySC remaining associated with the hub and a distally located post-mitotic daughter somatic cyst cell (SCC) [33]. Two SCCs enclose each gonialblast forming a testicular cyst “sealed” from the outside by the extracellular matrix (ECM) (Fig. 1) [74]. The gonialblast divides mitotically four more times to give rise to 16 interconnected spermatogonial cells, which then undergo pre-meiotic DNA replication, become spermatocytes, turn on the transcription program for terminal differentiation and undergo meiosis. During pupal stages testis morphogenesis is completed with the addition of the acto-myosin sheath originating from the genital disc [50]. The SCCs co-differentiate with the germ cells they enclose, grow enormously in size, elongate and accompany them throughout their differentiation steps up to individualization and sperm production in the adult testis [32].

### 2.1 Specification of the testis stem cell niche

Specification of the hub cells is a prerequisite for establishment of the testis stem cell niche per se. Hub cells are somatic cells specified before gonad formation from a subpopulation of the lateral mesoderm, the somatic gonadal precursor cells (SGPs), in bilateral clusters of the abdominal parasegments 10 to 13 [4,22,53,68,72,76]. The different SGP populations joining the embryonic male gonad orchestrate testis morphogenesis at this initial stage, since the germ cells represent a uniform population at this time. *zinc-finger homeodomain 1* (*zfh-1*), a key player in SGP specification, is initially expressed in cell clusters of the lateral mesoderm (PS2-14) whereas at a later stage *zfh-1* expression in parasegments 10-13 correlates with the specification of these cells as SPGs [9,68,92].

However, not only the hub cells but also the cyst cells are specified from the SGPs. The common origin between the hub and CySCs has been shown by lineage tracing experiments [25]. Hub cell fate vs. cyst cell fate is specified prior to gonad coalescence in a subset of SGPs upon Notch signaling activation [25]. Specification of CySCs vs. hub cell fate is further shaped by the antagonistic function of the cytoplasmic protein lines (Lin) and the transcription factor Brother of odd with entrails limited (Bowl) [40,111]. Bowl promotes hub cell fate and lines CySCs fate, evidenced by fewer hub cells in *bowl* mutant gonads and increased number of hub cells in *line*s mutant gonads. Also, *line*s depleted CySCs acquired some hub-like properties and markers [25]. This is further supported by the fact that both cell types can be traced with the same cell markers such as Zfh-1 and Traffic Jam (TJ) [111]. In the posterior SGPs, the epidermal growth factor receptor (EGFR) represses hub formation and allows its formation only at the anterior part of the gonad [46].

**Fig. 2:**
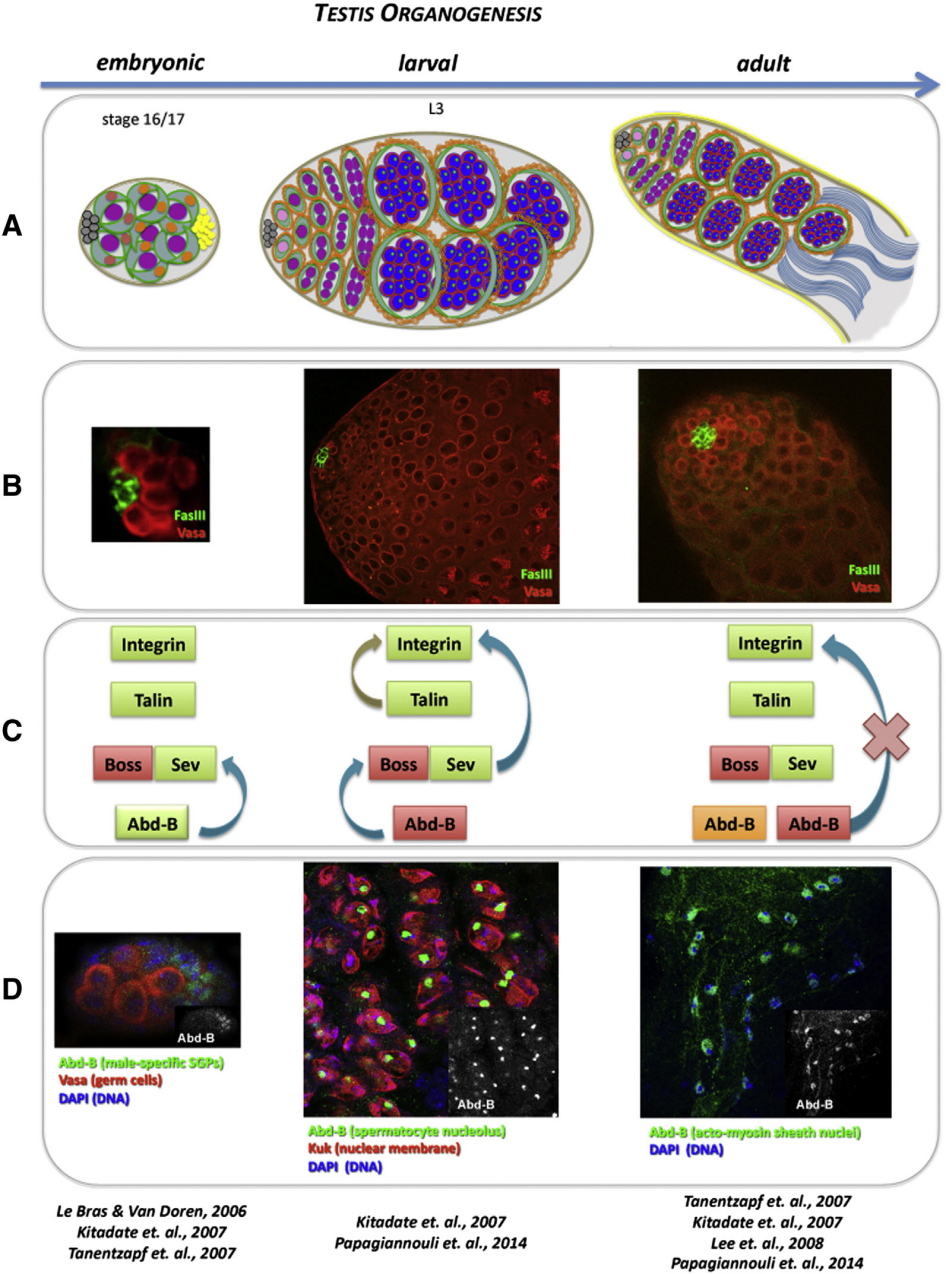
Regulation of stem cell niche positioning during progressive testis organogenesis. (A) Upper panel represents a schematic diagram of the embryonic male gonad, larval and adult testes during *Drosophila* testis organogenesis. Germ cells are shown in purple. Gray circles represent hub cells in the anterior of male gonads and testes. The somatic lineage (somatic gonadal cells or CySCs and SCCs) is shown in green. Within the male gonad, *abd-A* is expressed in red-colored nuclei, *abd-A* and *Abd-B* are co-expressed in orange-colored nuclei. Male-specific somatic gonadal cells expressing Abd-B are shown in yellow. (B) Confocal pictures, of an embryonic male gonad, larval and adult testes, showing the hub cells of the stem cell niche stained for FasIII (green) and the germline stained for Vasa (red). FasIII and Vasa staining of *Drosophila* embryonic gonads (left column, second panel) is reproduced from [53] (Elsevier & Copyright Clearance Center's RightsLink service; License Number: 3511390160410). (C) The players that control hub positioning and integrity in embryonic (left column), larval (middle column) and adult (right column) stages are shown: integrin [54,95], talin [95] and Sev [47,74] are required in the somatic linage (highlighted in green) and Boss [47,74] in the germline (highlighted in red) during all stages of testis organogenesis. Abd-B regulates hub positioning and integrity in all stages but from a different cell type in each stage: somatic gonadal. Arrows show established interactions among the players. Arrows of the same color connect factors belonging to the same pathway. (D) Lower panel confocal pictures show Abd-B localization in posterior somatic gonadal cells (left column), larval spermatocytes (middle column; Kuk marks the nuclear membrane) and adult acto-myosin sheath cells (right column). Small inset pictures show Abd-B localization only. L3: 3rd instar larval testis. In the lower panel, the reference list brings together the original research papers describing the role of each player in testis stem cell niche positioning and for each developmental stage (summarized in panel C).

Before gonad coalescence, the *Hox* genes *abdominal-A* (*Abd-A*) and *abdominal-B* (*Abd-B*) pattern the anterior-posterior (A/P) axis of the male embryonic gonad (Fig. 2): *Abd-A* specifies the anterior most SGPs giving rise to the hub, a combination of *Abd-A* and *Abd-B* specifies the posterior SGPs, and *Abd-B* alone specifies the male-specific SGPs [4,20,21,53]. Thus, *Abd-A* and *Abd-B* pattern the A/P axis of the formed gonad. Once specified, the hub cells are able to recruit the anterior-most germ cells to become the GSCs [88], giving rise to the male stem cell niche [11].

### 2.2 Testis stem cell niche positioning: state of the art

Stem cell niche and subsequent testis morphogenesis is a stepwise process based on the physical contact and diffusible signals exchanged between the germline and somatic cell populations [107]. In order to ensure normal niche function the hub cells of the *Drosophila* testis not only need to be properly specified but also need to be correctly placed and the architectural integrity of the system has to be maintained. Proper niche function in terms of hub positioning and integrity is tightly coupled to adhesion and cell communication, with βPS-integrin (encoded by the *myospheroid* (*mys*) gene) and the bride of sevenless (Boss)/sevenless (Sev) signaling pathway playing key roles in embryonic [47,95] and adult stages [47,54,74,95]. Integrin-mediated adhesion is important for maintaining the correct position of the embryonic hub cells during gonad morphogenesis (Fig. 2). In the absence of integrin-mediated adhesion, the hub cells still form a cluster, but instead of remaining at the anterior part of the gonad they migrate to the middle part of the developing gonad [95]. Disruption of integrin-mediated adhesion in adult testes, for example by knocking down *talin*/*rhea*, a gene coding for an integrin-binding and essential focal adhesion protein of the integrin-cytoskeleton [18,96], results in gradual hub disappearance, a phenotype which becomes more severe as adult males age [96]. As the hub is progressively lost in *talin*-depleted adult testis, the signals that normally emanate from the hub to instruct stem cell renewal are absent, driving the balance between stem cell maintenance and differentiation towards more differentiation. As a result the GSCs are progressively lost [95]. A similar hub displacement phenotype is observed by reducing Lasp levels, an actin-binding protein, in the adult testis [54]. It is known from vertebrate systems that Lasp interacts genetically with integrin [93] and in blood platelets Lasp requires integrin for its proper localization to the cytoskeleton [98]. In a few cases, loss of Lasp leads to hub integrity defects in which the hub cell arrangement is disturbed or double hubs are observed. Genetic interaction studies showed that βPS-integrin and Lasp proteins are active in different pathways, which cooperate to position the hub in adult testes [54].

The Boss/Sev pathway plays an important role in hub positioning and integrity in the *Drosophila* male gonads by preventing ectopic niche differentiation in the posterior gonadal somatic cells. Sev is activated by the Boss ligand emanating from the primordial germ cells to repress ectopic hub differentiation [47]. Upstream of this cascade, *Abd-B* activates *sevenless* (*Sev*) in the posterior male-specific SGPs (Fig. 2) [47]. Consistent with this observation, weak *Abd-B* mutant alleles result in hub expansion and hub integrity defects in embryonic gonads [53]. Boss and Sev are required for hub positioning and integrity in the adult testis, but the mechanism of action remains so far unknown. Taken together, hub positioning and integrity rely on the employment of the same players during testis organogenesis from embryonic up to adult stages of *Drosophila* development. However, the mode of action of these genes, their putative cooperation and stage-specific function remain so far elusive.

## 3 Stage and cell-type specific control of testis stem cell niche positioning and architecture

The establishment and maintenance of niche architecture is crucial for keeping the niche microenvironment and tissue homeostasis throughout adult life. Niche architecture relies largely on the physical interactions of the stem cell membrane with tethering molecules on neighboring non-stem cells or surfaces that keep niche integrity and allow the exchange of signals shaping the niche [84]. Therefore, basic molecular features of niche integrity are found to be common in different stem cell systems. One example is the interplay of integrins with focal adhesion proteins [91], the extracellular matrix (ECM) [34–36,87] and the guanine nucleotide exchange factor Vav [42,81], which is critical for retaining spermatogonial stem cells in the mammalian testis [45].

*Drosophila* testis organogenesis progresses through successive developmental steps (Fig. 2). A critical question is how the niche stays functional after its initial specification, as testis morphogenesis proceeds with the addition of new cell types from embryonic, larval and pupal stages to the mature adult testis. Moreover, despite the progress made in understanding niche architecture and positioning [47,53,54,95], until recently little was known about the upstream regulators providing positional cues to the downstream adhesion and cytoskeletal components.

### 3.1 Same players, different mechanisms: how the Abd-B and Boss/Sev cross talk controls larval hub positioning and integrity

Recent work revealed a new role for the posterior *Hox* gene *Abd-B* in niche positioning and integrity during larval stages (Fig. 1). Abd-B localizes in the nucleolus of germline spermatocytes in larval testis [74], and qPCR experiments indicated the presence of both *Abd-B m-* and *r-* isoforms in equal levels (F. Papagiannouli & I. Lohmann unpublished data). In addition to its described role in the male embryonic gonads, sustained activity of Abd-B in the pre-meiotic germline spermatocytes of larval testes is required to maintain niche positioning and integrity by regulating βPS-integrin and actin localization in the neighboring SCCs [74]. Cell-type specific knockdown of *Abd-B* in larval *Drosophila* germline spermatocytes, using an *Abd-B^RNAi^* transgene, leads to hub mispositioning, hub integrity defects and less frequently to the formation of two independent niches.

*Drosophila* bride of sevenless (Boss) is a G-protein coupled receptor membrane protein that was first identified as a ligand of the sevenless (Sev) receptor tyrosine kinase involved in eye differentiation. Previous studies in the eye showed that upon binding of the transmembrane protein Boss to its receptor Sev, Boss from the “donor cell” becomes internalized in the *Sev*-expressing “receiving cell” [10,51,52]. However, in the fat body Boss acts as a receptor and in response to glucose stimulation by a yet unidentified ligand, Boss becomes enclosed in internalized vesicles in the *Boss*-expressing cell [49]. This means that Boss has properties of both a ligand and a receptor. Endocytic membrane trafficking plays a critical role in GPCR signaling and regulation [106]. Upon ligand binding, GPCRs undergo conformation changes, cannot bind to G-proteins anymore, become phosphorylated by GPCR kinases and get endocytosed in clathrin-coated pits in a dynamin dependent manner, leading to numerous physiological outcomes including receptor desensitization (inactivation), resensitization (receptor recycling back to the membrane) or proteolytic degradation in lysosomes.

In the *Drosophila* testis, Boss is found in the germline (the spermatocytes), primarily in vesicles, whereas Sev localizes in the SCCs enclosing them. Analysis of the role of the Hox protein Abd-B in the *Drosophila* testis revealed that Abd-B present in spermatocytes acts upstream of the Boss/Sev pathway to regulate hub positioning and integrity, which finally leads to loss of integrin localization in the SCCs [74]. Abd-B performs its function by affecting Boss internalization in the germline (*Boss*-expressing cell), as Boss is lost from internalized vesicles in *Abd-B* depleted testes. Expression of activated Sev in cyst cells of Abd-B depleted testes can fully rescue the phenotype, meaning that Sev is downstream of both Abd-B and Boss. This means that in testicular cysts Boss acts as the ligand of Sev that signals from the “donor” spermatocyte cell to the “receiving” cyst cell when at the same time Boss possesses GPCR properties as immunostainings have shown that Boss gets enclosed in internalized vesicles in the “donor cell” [74]. This is further supported from the fact that expression of *dynamin*/*shibire*, required for clathrin-mediated receptor endocytosis from the plasma membrane [67,101], in spermatocytes of *AbdB* depleted testes could partially rescue hub positioning and integrin localization in cyst cells [48,78]. This observation suggests that Boss functions, similar to other GPCRs, in a dynamin-dependent way for its endocytosis in trafficking vesicles in spermatocytes. However, the precise mechanism of Boss action in the spermatocytes upon binding to Sev that guides germline-to-soma communication, and upon Boss endocytosis in spermatocytes, is not fully understood.

In order to elucidate how the Hox transcription factor Abd-B affects Boss localization, genes directly regulated by Abd-B in the *Drosophila* testis were identified by mapping Abd-B binding sites in vivo using the DNA adenine methyltransferase identification (DamID) technology [13,102,103,105]. This analysis resulted in the identification of Abd-B binding regions in larval testes and the associated genes [74]. Since Abd-B controls signaling between the germline and somatic lineage by regulating genes required for Boss receptor recycling or trafficking, further analysis focused on genes involved in trafficking processes. Two genes, one encoding the non-receptor tyrosine kinase Src oncogene at 42A (Src42A) and another one encoding the putative signal recognition binding protein Sec63, were identified as potential mediators of Boss function in the larval testis (Fig. 1B). Src42A is a non-receptor tyrosine kinase [94] implicated in many signal transduction pathways [109], cell signaling coupled to integrin-related scaffolding proteins [1,97,99], modulation of the actin cytoskeleton [65,94] and maintenance of adherens junctions [89]. Notably, vertebrate Src family members are important for the internalization of G-protein coupled receptors [37,58]. On the other hand, the ER membrane protein Sec63 has been shown in yeast and mammals to be important for the biogenesis of secretory and membrane-bound proteins by regulating the translocation of nascent proteins into the ER [44,60]. In support of a direct regulatory interaction between Src42A and Abd-B in the larval testis, *Src42A* and *Sec63* mRNA levels were significantly reduced in spermatocytes of *Abd-B* depleted testes [74]. Functional analysis confirmed that *Src42A* and *Sec63* depleted testes mimic the loss of *Abd-B* function as Boss protein was not detected in vesicles, the hub was mispositioned and βPS-integrin was not properly localized in SCCs. Based on these experimental data we postulate that Sec63 regulates Boss protein translocation and maturation in the ER while Src42A affects Boss endocytosis and receptor recycling.

Taken together, Abd-B acts as an upstream regulator of the Boss/Sev pathway in this system, by controlling *Sev* expression in the embryonic male gonad [47] and Boss function in the larval testis via *Sec63* and *Src42a* expression [74]. Abd-B seems to regulate germline-to-soma signaling in a very precise and fine-tuned manner by controlling several critical steps in the signaling events, like the biogenesis and recycling of signaling components. In particular, the switch of Abd-B expression from the embryonic male-specific somatic cells to the larval spermatocyte germ cells (Fig. 2) correlates with a change in the Abd-B dependent mechanism of hub positioning between embryonic and larval stages.

### 3.2 The AbdB - Boss/Sev - integrin cascade: cell autonomous and cell non-autonomous effects in larval stem cell niche positioning and function

#### 3.2.1 Cell autonomous effects: the role of integrin-mediated adhesion

Although we have uncovered the mechanic link of Abd-B to the Boss/Sev signaling in the larval testis that drives germline-to-soma communication and integrin localization in the neighboring cyst cells [74], the role of downstream effectors in hub positioning and integrity within the cyst cells such as integrin-related and focal adhesion proteins has not yet been addressed. Integrins are dynamic cell-surface receptors, composed of one α- and one β- subunit, that provide a link between the ECM and the actin cytoskeleton [6,23,104]. On the cytoplasmic side of the cell membrane, integrins assemble into large multi-protein complexes composed of integrin-binding adaptor proteins, focal adhesion scaffolding proteins, actin-binding proteins and cell-signaling molecules [6,24,26,39,104].

In order to address the potential role of such factors in testis stem cell niche positioning and integrity, we characterized their localization and function in the *Drosophila* larval testis. Talin, encoded by the *rhea* gene in *Drosophila*, is involved in integrin-mediated adhesion and in hub positioning and maintenance in the adult testis [95]. In the larval testis, talin co-localizes with βPS-integrin in CySCs and SCCs, and *talin* depleted testes display defects in niche positioning and hub integrity. Consistent with its described function in adult hub positioning [54], Lasp is required for larval hub positioning as well. Similar results were obtained with Vav, a guanine nucleotide exchange factor (GEF) and activator of the Rho-family of proteins [100]. Its vertebrate homologue Vav1 controls integrin clustering at immunological synapses [42] and regulates perivascular homing and bone marrow retention of hematopoietic stem cells and progenitor cells [81]. In the *Drosophila* testis, Vav not only associates with the epidermal growth factor receptor (EGFR) in the SCCs to regulate Rac1 activity [82] but co-localizes with βPS-integrin and affects larval niche positioning and integrity.

Consistent with the association of hub positioning defects with hub architectural abnormalities, cell-type specific RNAi knockdown of *focal adhesion kinase* (*Fak56D*) and *Paxilin* (*Pax*) in larval cyst cells leads to hub shape and integrity defects whereas knocking down *pinch* (also called *steamer duck*/*stck*) leads to hub-split and formation of two independent stem cell niches. On the extra-cellular space, ECM proteins such as Laminin A [30,59] and the Laminin A-type protein Wing blister (Wb) [63], co-localize with βPS-integrin and affect larval niche positioning [74]. Taken together, integrin in association with focal adhesion and adaptor proteins on the intracellular side, and ECM on the extracellular side of the somatic lineage (CySCs and SCCs) orchestrate multiple adhesions to position the niche and preserve its architecture. Analysis of the genetic interactions of *Abd-B*, *Boss*, *Sev* and *talin* with *βPS-integrin^mys^* revealed that larval hub positioning depends on two independent pathways (Fig. 1B), one controlled by the AbdB-Boss/Sev cascade and the other mediated by talin, both of which converge at integrin that acts as a central player in this system.

#### 3.2.2 Cell non-autonomous effects: positioning the larval hub

We have shown that local Boss/Sev signaling, mediating germline–soma communication within the testicular cysts, is required for βPS-integrin localization in the cyst cells. Previous work identified integrin as central downstream effector gene in hub positioning and architecture of the *Drosophila* testis [74,95]. Integrin adaptor proteins such as Paxilin and *Drosophila* focal adhesion kinase (Fak56D) act in a similar way, since knocking-down these genes in the hub, CySCs and SCCs of the larval testis results in hub positioning and integrity defects (F. Papagiannouli & I. Lohmann unpublished data). Interestingly, all the above mentioned factors are expressed and exert their functions outside the hub: Abd-B and Boss in the germline, Sev, integrin, talin, Lasp, Vav and Wb in larval testis cyst cells [74]. One of the emerging questions is how these players act on stem cell niche positioning and integrity outside of the hub region. Our current hypothesis is that integrins, ECM components, focal adhesion proteins and actin filaments together build a dynamic scaffolding network thereby regulating male stem cell niche positioning and integrity in a cell non-autonomous manner. As the role of integrins and their binding proteins in mechanosensitive adhesion is well established [16,41,64,70], we hypothesize that integrin-mediated adhesion in cyst cells generates tensional forces through interaction with the cytoskeleton and ECM to maintain the testicular cysts and overall testis rigidity, which is required for keeping the hub at the anterior part of the testis outside the integrin expression region. This is achieved by a two-step process: first, local signaling between the differentiating germline and the neighboring SSCs, that form together a functional unit called cyst, ensures proper integrin localization in the individual testicular cysts (Fig. 1B). Subsequently, integrin interacts with actin, the cytoskeleton and the ECM that provides the testis with the rigidity required for keeping the hub at the anterior part of the testis (Fig. 1A). The Hox transcription factor Abd-B is a crucial player in this network, since it provides critical inputs for the main components of this regulatory cascade.

#### 3.2.3 Cell non-autonomous effects: asymmetric germline stem cell division and testis homeostasis

An essential feature of stem cell niches is the regulation of stem cell renewal, which is often coupled to asymmetric stem cell division. The mechanisms underlying asymmetric stem cell division, such as the establishment of spindle polarity, cell polarity or segregation of stem cell determinants, often involve overlapping subsets of factors [31]. Integrins play a central role not only in anchoring the stem cells to the niche but also in establishing polarity in niche architecture. For example, integrin-mediated adhesion is implicated in centrosome function and polarization, microtubule assembly [15,27] and asymmetric cell division in human and ferret neocortex [28]. If niche architecture and integrity are compromised, age-related changes, like the reduction in stem cell number and activity, lead to failure in tissue homeostasis [108].

Interestingly, loss of Abd-B in larval germline spermatocytes and subsequent loss of βPS-integrin from the neighboring cyst cells is associated with cell non-autonomous effects in the GSCs surrounding the hub: GSC centrosomes are frequently mispositioned and GSC division rates are reduced, which results in a dramatic decline of pre-meiotic cell populations in adult testes. Presumably, the delay in the GSC cell cycle leads to reduced spermatogenesis over the larval, pupal and adult stages and to the accumulation of defects normally observed in aged testes [5,12,108]. In most stem cell systems including *Drosophila*, aging is associated with progressive loss of stem cell number and activity, leading to compromised tissue function and regeneration [108]. Along this line, niche architecture and integrity, that provide the extrinsic physical and self-renewal cues to the stem cells, are also associated with age-related changes [73,108]. Although it is not yet resolved whether the cell non-autonomous effects in the GSCs in *Abd-B* depleted larval testes result from a compromised integrin function or from the hub displacement per se, the effects on GSC centrosome positioning, division rate and on adult pre-meiotic stages, emphasize the importance of continuous inputs that maintain niche function and architecture throughout testis organogenesis.

In sum, Abd-B acts as an upstream regulator of integrin-mediated adhesion in order to maintain the niche architecture, ensure proper niche and GSC function, and prevent the accumulation of aging-related effects. The new insights into the role of Abd-B in the *Drosophila* male stem cell niche, underline the requirement for proper niche positioning and architecture, link tissue architecture to intrinsic positional cues and support the view that *Hox* genes act not only as major developmental switches but also as cell-type switches, as they are turned on and off in a cell-type specific manner during development.

### 3.3 Different spatial pattern, same function: the cell-type and stage specific role of Abd-B

So far we have shown that niche positioning and architecture are tightly coupled to niche function and maintenance during testis organogenesis. A great number of studies as well as our own data have shown the critical requirement of integrin, talin, Boss and Sev for proper hub positioning and integrity throughout all stages of testis organogenesis [47,54,74,95]. Lasp requirement has been described for the adult stages [54] and cell-type specific knockdown of *Lasp* in the somatic lineage of the larval testis confirmed its requirement throughout testis organogenesis (F. Papagiannouli & I. Lohmann unpublished data). Interestingly, these players exert their function always from the same lineage: Boss from the germ cells (embryonic pole cells or larval spermatocytes), integrin, talin, Sev and Lasp from the somatic lineage (somatic gonadal cells or testis cyst cells).

But how does the niche cope with the addition of new cell types as testis organogenesis proceeds from embryonic, larval and pupal to the mature adult stages? Several lines of evidence indicate that niche architecture and function is cell-type and stage specifically regulated and that a factor can change its activity at different developmental stages (Fig. 2). One such example is the Hox transcription factor Abd-B: in the embryonic male gonads, Abd-B regulates *Sev* expression from the male-specific somatic cells [47], while in the larval testis Abd-B regulates the function of Boss via *Sec63* and *Src42A* expression, from the germline spermatocytes [74]. In the adult testis, *Abd-B* is expressed additionally in the nuclei of the acto-myosin sheath [Fig. 2D; [74]], which surrounds the adult testis and fuses it to the seminal vesicle. Recent work provided evidence that in contrast to the larval stages, Abd-B from the adult testis spermatocytes no longer affects integrin localization in the neighboring SCCs [74], meaning that Abd-B has most likely other germline stage-specific functions in the adult testis spermatocytes. Interestingly, knocking down Abd-B in the adult testis acto-myosin sheath, via cell-type specific RNAi or mosaic clones, affects the positioning of the hub and overall testis rigidity (F. Papagiannouli & I. Lohmann unpublished data). Therefore, the switch of Abd-B expression from the embryonic male-specific somatic cells to the larval spermatocyte germ cells and the adult acto-myosin sheath correlates with a change in the Abd-B dependent mechanism of hub positioning during subsequent steps of testis organogenesis. Furthermore, this also shows that a combination of critical players and pathways is employed and continuously provides inputs essential for maintaining the function of the stem cell niche. This underlines the vital importance to preserve adult germline stem cell function, protect spermatogenesis and produce healthy gametes and progeny critical for organismal function. Consistent with the newly emerging theme in the Hox field, Abd-B acts as precise cell-type specific upstream micromanager of male stem cell niche architecture, positioning and function, which is required continuously as the testis matures and becomes a functional part of the male genitalia to finally produce gametes.

## 4 Summary and outlook

Male stem cell niche function in *Drosophila melanogaster*, from initial specification to its continuous maintenance during testis organogenesis and adult life, is a dynamic process that relies on a combination of upstream regulators and a network of downstream realizators. The organism employs a number of factors and parallel pathways to protect the function of the stem cell niche. Notably, correct niche positioning and architecture are a prerequisite for stem cell niche function in order to prevent the accumulation of aging-related defects in testes at adult stages when reproduction starts. The Hox transcription factor Abd-B is a crucial cell-type specific upstream regulator in this network, since it provides critical positional cues for the main components of this regulatory cascade. In addition to its early role in the embryonic male gonadal cells [4,21,53], sustained activity of the Hox transcription factor Abd-B throughout larval stages and adulthood is required to maintain the position and architecture of the male stem cell niche. Thus, our findings are consistent with a newly emerging theme in the Hox field, namely that *Hox* genes act directly not only early in the specification of certain cell types but also in later events when these cells mature, become functional and form complex structures, like the neuromuscular junction or the testis, which need to be maintained.

As several of the main players analyzed in the *Drosophila* testis are conserved in other systems, related functions have been demonstrated in different organisms and tissue contexts. The vertebrate *Abd-B* homologues within the *Hoxa* and *Hoxd* clusters are necessary for patterning the genitalia: combined *Hoxa13* and *Hoxd13* inactivation results in agenesis of external genitalia [14], whereas *Hoxa10* homozygous mice show cryptorchidism, spermatogenesis defects and increased sterility [77,83]. Thus, *Abd-B* homologues have related functions in male spermatogenesis, genitalia development and fertility. Similarly, integrins have been implicated in stem cell maintenance and niche function in multiple tissues [7,26,38,56,75,86,110]. In particular, α6 integrin is a well-established surface marker for spermatogonial stem cells in mammalian testes [90], whereas the interplay of β1-integrin with focal adhesion proteins regulates movement of the germ cells across the seminiferous epithelium [91]. Moreover, β1-integrin in spermatogonial stem cells and Sertoli cells plays an important role in “homing” spermatogonial stem cells at the basal membrane, which is critical for male fertility restoration and spermatogenesis regeneration [19,45,71]. Presumably, despite the differences in niche architecture across different stem cell systems, homologues or equivalent factors are frequently utilized for the execution of analogous tasks. Accordingly, the mechanisms of stem cell niche function and spermatogenesis we discover in *Drosophila* could be used as a paradigm to understand similar regulatory strategies occurring in other stem cell systems and organisms.

Comparable to the cascade uncovered in the *Drosophila* testis, the central morphogenetic function of Abd-B is also illustrated in the development of structures such as the posterior spiracle and the genital disc [17,29,57]. In the *Drosophila* embryo, Abd-B directly controls the expression the early-transcription and signaling molecules *spalt* (*sal*), *cut* (*ct*), *empty spiracles* (*ems*) and *unpaired* (*upd*), which in turn activate a battery of downstream cell adhesion, cytoskeleton and cell polarity “realizator” genes leading posterior spiracle morphogenesis [57]. In this way, Abd-B as an upstream regulator activates a genetic cascade of direct and intermediate regulators that coordinate the local cell-specific behaviors and confer the morphogenetic properties sufficient for spiracle organogenesis [57]. Another characteristic example is how *Hox* genes and sex-determination direct the development of female and male genitalia [29,80]. Both sexes of *Drosophila* have a single genital disc formed by the primordial A8, A9 and A10 abdominal segments. The female genitalia develop from the A8 and the male from the A9 segments. In the embryonic as well as in the larval genital discs, the isoform Abd-B^m^ is expressed in A8 for the development of female derivatives and the isoform Abd-B^r^ in A9 shapes the male genitalia. Cross-regulatory interactions between abd-A, Abd-B^m^ and Abd-B^r^ shape the internal female genitalia, so that Abd-B maintains *abd-A* transcription in contrast to the embryonic epidermis where Abd-B represses *abd-A* [29]. Moreover, Abd-B^m^ is needed for the external female genitalia and Abd-B^r^ for the male genitalia. Finally, the Abd-B^m^ isoform regulates the clockwise (dextral) looping of male genitalia, a typical left/right asymmetry feature mediated by its direct target *myosinID* [17]. Therefore, Abd-B isoforms control the morphogenesis of female and male genitalia in a cell- and stage-specific way. All the examples from *Drosophila* discussed here, emphasize the micromanager role of Abd-B, which through complex genetic interactions guides morphogenetic events as diverse as male stem cell niche positioning and maintenance, posterior spiracle organogenesis and genitalia shaping. This raises the possibility of organogenesis being a common feature of Abd-B and likely a common property of Hox proteins in general.
